# What is the Modern Human Eating? Dietary Transition of the Age-Old to the Modern Man of India

**DOI:** 10.3389/phrs.2022.1604058

**Published:** 2022-03-18

**Authors:** Daisy A. John, Giridhara R. Babu

**Affiliations:** ^1^ Indian Institute of Public Health, Bengaluru, India; ^2^ Department of Population Medicine, College of Medicine, QU Health, Qatar University, Doha, Qatar

**Keywords:** food transition, processed food, globalization, staples, behavioral change

## Abstract

**Objectives:** The objective of this review is to examine the changes in food consumption pattern of Indians over the years and to recommend evidence-based policy making regarding all the factors affecting food consumption.

**Methods:** We have reviewed the articles from major databases such as PubMed and Google Scholar. The keywords used for the search included dietary pattern, dietary trend, dietary intake, food system, nutrition system, prehistoric food systems, drought, famine, whole grains, diets, prices, income, environment, urban food consumption, processed foods, food security, food preferences, demographic transition, fat intake, food production, public distribution system, food consumption pattern, Indian agriculture, and India.

**Results:** There is no facilitating environment for the production and cultivation of healthy and sustainable food.

**Conclusion:** Policymakers should make major amendments to food and agricultural policies, and demotivate the consumption of junk food.

## Introduction

The food pattern of human beings has been evolving since the time it is recorded. The objectives of this review are to understand how the food consumption pattern changed in India over a large timeline and the second objective is to understand how home-made food and food available outside has changed, and how industry-manufactured food penetrated households gradually. Food pattern can be defined as the quantities, proportions, variety, or combination of different foods and drinks in diets and the frequency with which they are habitually consumed [1]. There are five stages in nutrition transition [2]. These are the stages of food gathering, famine, receding famine, degenerative disease (non-communicable disease), and behavioral change; most countries are in stage 3 currently [2]. Human habitat during the early hunting-gathering period was on hilly, rocky, and forested regions and this had sufficient plant and animal food resources, both land and aquatic, and grinding stones were used for processing plants such as wild rice. During this period, there was sufficient food for people in the country. Next was the phase of agriculture, which took place around 8000 years ago. After the advent of agriculture, humans shifted from the Hilly and rocky regions to the alluvial plains, which had fertile soil and perennial availability of water. In India, the introduction of iron technology helped the farmers to clear the dense and tangled forests of the middle and lower Ganga plains, which was one of the first locations of civilization in India [3]. The hunter-gatherers had a different diet as compared to the agriculturalists. The agriculturalists lacked food diversity as they cultivated particular crops and consumed those, whereas the hunter-gatherers as the name suggests, roamed around, hunted, gathered, and consumed diverse food items [4].

Later due to droughts and crop failures, India had a long history of famine which led to starvation and deaths of around 2 million people [3, 5]. This was complicated by a rising population, lack of irrigation, and low crop yields. The method of irrigation was introduced only in the 20th century [6]. Periods of famine occurred in India in the years 1769−70, 1783−84, 1791−92, 1837−38, 1860−61, 1865−67, 1868−70, 1873−74, 1876−78, 1896−97, 1899−1900, and 1943−44 [7]. The primary and most talked about of all the famines is the Bengal famine, which happened during the war period, due to shortage of rice production caused by the epidemic of helminthosporium disease which attacked the rice crop. The price of commodities rose and was not affordable for the common people. Rural to urban migration increased for employment and/or food, and people who could not find those died of starvation [5]. A lot of people lost jobs as there was not enough food production [8].

**FIGURE 1:**
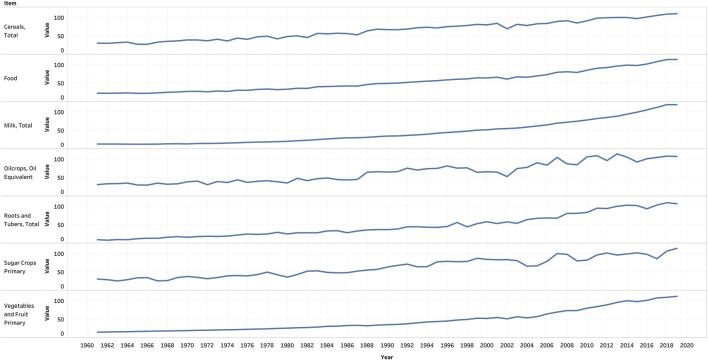
Production pattern of food groups over the years, FAOSTAT, Food and Agricultural Organisation, 2021.

Later, the advent of the Green revolution resulted in increased production of food items by increasing the farming area, double-cropping, adoption of high-yielding variety seeds, use of chemical fertilizers and pesticides, improved irrigation facilities, improved farm equipments, and crop protection measures, etc. Although these kept a check on famine and malnutrition. these were unsustainable measures of food production [9]. Figure 1 shows the production pattern of various food groups over the years [10]. Overall food production has increased over time, and the period following the Green revolution saw an increase in the production of all kinds of food groups. India also saw a White revolution where India emerged as the largest producer of milk; further, India also became the second-largest producer of fruits and vegetables. The agricultural trade in India also increased, i.e., the net agricultural exports increased from $2.7 billion in the period 1990-1991 to around $10.7 in the year 2008–2009. India managed to export around 33.2 million tons of rice during the period from 2001 to 2002 and 2008-2009.

## Methods

The topic under consideration requires a literature review because nutrition transition is a phenomenon that is actively happening in India. To understand the factors in various fields which facilitated the nutrition transition, a literature review would help in generating the most appropriate recommendations. The search engines used for this review include PubMed and Google Scholar. The search strategy used in PubMed was as follows:

(((((((((((((((((((((((dietary pattern) OR (dietary trend)) OR (dietary intake)) OR (food system)) OR (nutrition system)) OR (prehistoric food systems)) OR (drought)) OR (famine)) OR (whole grains)) OR (diets)) OR (prices)) OR (income)) OR (environment)) OR (urban food consumption)) OR (processed foods)) OR (food security)) OR (food preferences)) OR (demographic transition)) OR (fat intake)) OR (food production)) OR (public distribution system)) OR (food consumption pattern)) OR (Indian agriculture)) AND (“India”).

The search was done for the period of 1990 and 2021; review articles were selected and a total of 10,821 results were obtained. After screening the title and abstract, a total of 27 articles were found relevant to the topic. The keywords used for searching literature in Google Scholar were food transition, India, millets, and processed food in a free-hand search.

## Results

### Rise of Food Insecurity Issues

To deal with the rising issue of food insecurity, social safety nets and programs that can guarantee income generation are required. The Public Distribution System is one such program that distributes essential commodities such as rice, wheat, sugar, kerosene, etc. Other plans such as food coupons, social audits for identifying the poor, and also direct cash transfers, etc. are in the pipeline. The National Rural Employment Guarantee Scheme which started in the year 2005 aimed to provide a minimum of 100 days of guaranteed wage employment for the adults of a household who volunteered to be a part of this scheme. Similarly, the National Food Security Bill ensures that every family below the poverty line is entitled to 25 kg of wheat or rice per month for 3 Rs. per kg. But, despite all these efforts, nutritional security is not yet achieved in India [11].

Overall, in India, there has not been much improvement in the case of poverty status despite improvements in the Gross Domestic Product (GDP). Although the GDP growth rate has been above 5%, the percentage of Indians living in absolute poverty (defined as less than US$1.25 per day, in the year 2005, in purchasing power parity) reduced only by 0.7% between the period 1994 and 2005. Essentially, in India, the economic improvements have not led to an improvement in human development indicators. Income inequality widened and the Gini coefficient which measures the rate of inequality increased from 28.6 to 30.5 in rural India and from 34.3 to 37.6 in urban India [12].

Currently, the question is not only food security, but also food diversity. The challenge is to produce sufficient food for everyone in an environmentally and financially sustainable manner and also increase the diversity in the food basket. Various flagship programs were initiated for this purpose. This included the National Agricultural Development Program, National Food Security Mission, and other programs such as the Accelerated Irrigation Benefit Program, Integrated Watershed Management Program, Micro Irrigation Mission, etc. China also saw the fastest rate of overall per capita GDP growth in the period after 1991 on the way to achieving food security. In the plans for improving nutrition and food security by ADB, it is mentioned that there is a lot of potential in the eastern side of India and that another Green revolution can be started there [11]. But, there have been severe impacts of the Green revolution previously which are extensively studied and were not sustainable practices. It is not ideal to resort to the Green revolution again. Such policy documents need a rework on what can be done in the future. What is required is the usage of existing traditional knowledge such as increasing the production of millets that are environmentally sustainable, less resource-intensive, and which can withstand drought, rather than beginning another Green revolution [9].

### Entry of Processed Food Into the Food System

After the famine and Green revolution, there was the onset of the industrial revolution in India and there was increased availability of processed food [6]. Food processing happens at various levels from mildly processed raw rice to highly processed refined-grain edible bread [13]. There was an increased preference for processed rice because of its better taste. Simultaneously, there was a substantial reduction in the consumption of traditional grains such as millets and indigenous varieties of rice [14]. This along with other factors such as a sedentary lifestyle led to an increase in the number of people with non-communicable diseases [15].

In the metro cities of India, people eat out from roadside eating places, tea and snacks shops, and street vendors. The process of eating out has become pervasive, and households with younger, single individuals spend more than 50 percent of their expenditure eating outside and particularly 10 percent on processed food [16, 17]. There is an increased consumption of biscuits, salted snacks, prepared sweets, edible oils, and sugar, and purchased foods in the last two decades in the urban areas. This ranges from 100–427 g/per capita/day and average consumption is about 167 g/per capita/day from the lowest to the highest class [18]. India is also famous for its traditional street food items, sold by hawkers and vendors at various places, particularly around schools. These items are comprised of deep-fried food such as samosas, chat, *tikkis*, and jalebis. These food items have a high amount of trans fat content [19]. This has become so pervasive and was facilitated after the Indian parliament identified that street vending of food is essential for the development of the country and passed the Street Vendors Act, to protect the rights of street vendors [20]. Another kind is instant food that can be prepared at home where the raw ingredients are available in packages [19].

### The Current Stage of Nutrition Transition in India

In India, the process of nutrition transition in the chronic diseases’ stage has further taken place in three stages [21]. This includes stage 1, where consumers moved away from traditional staples to a more westernized diet. This included more consumption of wheat in the form of bread, cakes as well as cookies. In stage 2, there were marked influences of globalization which most of the Indians are following currently. In this stage, there is an increased consumption of processed, ready-to-eat, deep-fried food, and food items with added preservatives. The advertising of unhealthy food items through audio-visual media further influences the dietary habits of children as well as adolescents in India, which leads to an increase in the prevalence of obesity. In stage 3, the stage of behavioral change, which is a reversal of diets, these people have a relatively healthier lifestyle and have improved eating habits. But, the last stage is mostly seen in the wealthier class of society. This is because this class owns economic resources sufficient to buy costly healthy foods, and avail themselves of exercise facilities, which includes expensive equipment as well as gymnasium visits [22].

### Impacts of Food Security in the Relationship With Migratory Patterns Within India

Although other countries have seen an improvement in food security with improvement in the economic status, in India, the improvement in food security has only been minimal and this rather unexpected phenomenon in India is referred to as the food security enigma [23, 24]. In India, economic growth is only urban-centered and not in rural India. This urban-centered nature of economic growth weakened agriculture as a source of income and food security in rural India. In addition to this, it is identified that around 42% of the population in India does not own agricultural land. Thus, the only resort for this population is to depend on daily wages. To achieve this, there is an internal migration of people from rural areas to urban areas. This migration is seen to have some impact on food security. The households which had at least one migrant saw a better food security status as compared to the households which did not have any migrant working elsewhere [24]. On the other hand, although there is some improvement seen due to migration, studies also prove that many migrants tend to follow unhealthy lifestyles and behavior patterns. These people are found to have increased consumption of saturated fats, lower consumption of complex carbohydrates, polyunsaturated fatty acids, fibers, and were also found to have lower levels of physical activity. The migrants were found to have a reduction in the consumption of cereals and an increase in the consumption of dairy products. These people were also found to have increased risk for cardiovascular disorders and hypertension, as compared to their rural counterparts [25]. Since the households live hand to mouth, they could never afford to miss even a single day’s work. Hence, the migrants face the issue of food insecurity as well as malnutrition. In addition to this, women are dependent on their husbands for food items to be bought for their homes, and most of the time, the husbands spend a lot of their income on alcohol. Hence, alcoholism is one of the main reasons of food insecurity in migrant households. Also, most of these workers travel far from home and are not able to carry home-made food and hence tend to eat outside food. The neoliberal perspective deems food security as food insufficiency; social justice and workers’ rights are not in the picture. Simultaneously, food security and food sovereignty are two different aspects which are to be considered, particularly in the context of migrant workers. According to Schanbacher, food security based on the model of globalization limits human relationships only to economic value, whereas the food sovereignty model is based on human relationships in terms of mutual dependence, cultural diversity, and respect for the environment. In the case of migrant workers, only the economic aspect is taken into account, whereas food sovereignty is based on accessing social justice, which would also ensure that it is the right of every human being to get access to nutritious and healthy food. The consideration of approaches leading to food sovereignty includes political environment, expansion of economic democracy, and transformation of unequal social relations. Ensuring food security means increasing the income or employment opportunities of the people living in poor conditions. To achieve food sovereignty, democratic social relationships of production with workers who control natural and state resources are required. It needs the community to control food production, circulation, and consumption, rather than the corporations controlling these [26]. Similarly, migrants are not entitled to food distribution systems such as the Public Distribution System. Hence, they are forced to buy food items at higher prices, and they are not able to afford fruits and vegetables [26].

### Changes in Dietary Diversity and Total Diet Intake in India

In a study conducted to calculate dietary diversity scores of Indian diets, it was identified that from 1993 through 1994, the household dietary diversity scores of rural households of 12 food groups over a 30-day recall period, was 9.08 and that of the urban households was 9.34. By the years 2011–2012, the score of rural households was 9.71 and that of the urban households was 9.57. Hence, the dietary diversity of the rural people has increased by 0.63 food groups, and not much change was seen in urban food groups [27].

The intake of cereals and millets reduced by a value of approximately 137g in a period of four decades. The study conducted by NNMB also found that people in rural areas in India had an inadequate diet and ate much less than the recommended levels. There was a notable decrease in the consumption of green leafy vegetables, pulses and fats, and oils in the households. There was a large decline in almost all nutrient intake in India [28].

### Policy-Level Measures Leading to a Change in Food Consumption Pattern

After the Green revolution in the years in late 1960s in Punjab, there was a tremendous decline in the cultivation of cereals such as millet and sorghum and a steady increase in the cultivation of rice and wheat [29]. The variation in the production of different kinds of food items over the years is shown in Figure 1 [10]. From the figure, it is evident that there is an increase in the production of certain vegetables, rice, and wheat. The unit of the items shown in the graph is in tons. People were provided heavy subsidies to produce new varieties of rice and wheat in India in the post-independence period [29]. The distribution of these new varieties of rice and wheat was mainstreamed through the PDS. Millets were not cultivated enough, and the government provided new varieties of rice, wheat, sugar, and kerosene through PDS to around 330 million people [30–32].

Further, as a modification of the PDS, the Revamped Public Distribution System (RPDS) started in 1992. This focused on the poorer sections of society and it allocated 10 to 20 Kg of food grains to BPL families at a 50% waived rate [30].

Agricultural policies formulated as early as the 1990s had the objectives of raising food availability by increasing food production and making it available to consumers. This was to be achieved by 1) using Minimum Support Prices (MSPs), through a regular and guaranteed procurement of specific food grains, 2) using open market operations to maintain price stability through different seasons of the year, 3) by maintaining a buffer stock of food grains, and 4) by using the Public Distribution System so that the food items can be made available to the public at a reasonable price to ensure food security, particularly for the low-income people. Around 1992, newer policies were focusing on liberalizing some of the restrictions set on international trade, after which most of the products could be imported to India without the need for a license. The State Trading Corporation removed quotas, reduced tariffs, and eased licensing agreements [33].

### Trade-Related Changes

To achieve national self-sufficiency, various adaptations were made in India in the years around the 1950s and there was import-substituting industrialization. This was done to reduce the dependence on external trade, particularly imports. But, these policies had various side effects which included booming of less-efficient high-cost manufacturing industries, and the necessity of subsidization of exports for high-priced imports to maintain competitiveness. All these started mounting pressure on the government demanding a necessity to liberalize many of the policies and even liberalization of imported items [33].

A statistically significant linkage was found between the trade reforms and the regional food consumption in rural India. The regions that were more exposed to tariff reductions are shown to consume relatively fewer cereals, more eggs, fish, and meat, more temperate zone fruits, and an increase in high-calorie food consumption [34].

**FIGURE 2:**
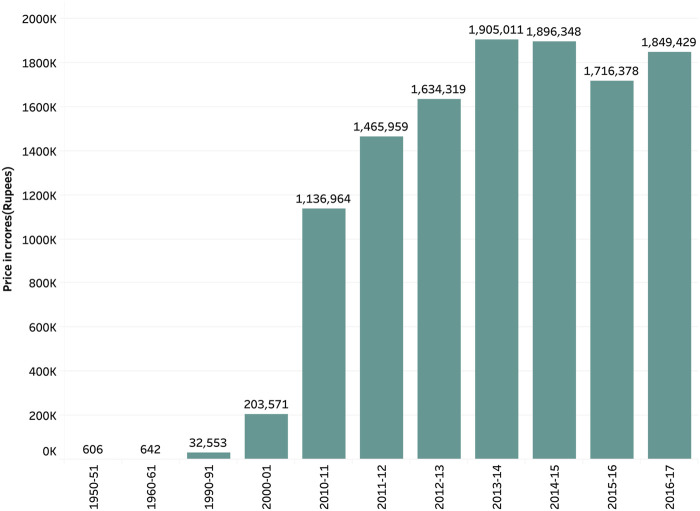
Exports of food items from India, Pocket book of Agricultural Statistics, India, 2017.

**FIGURE 3:**
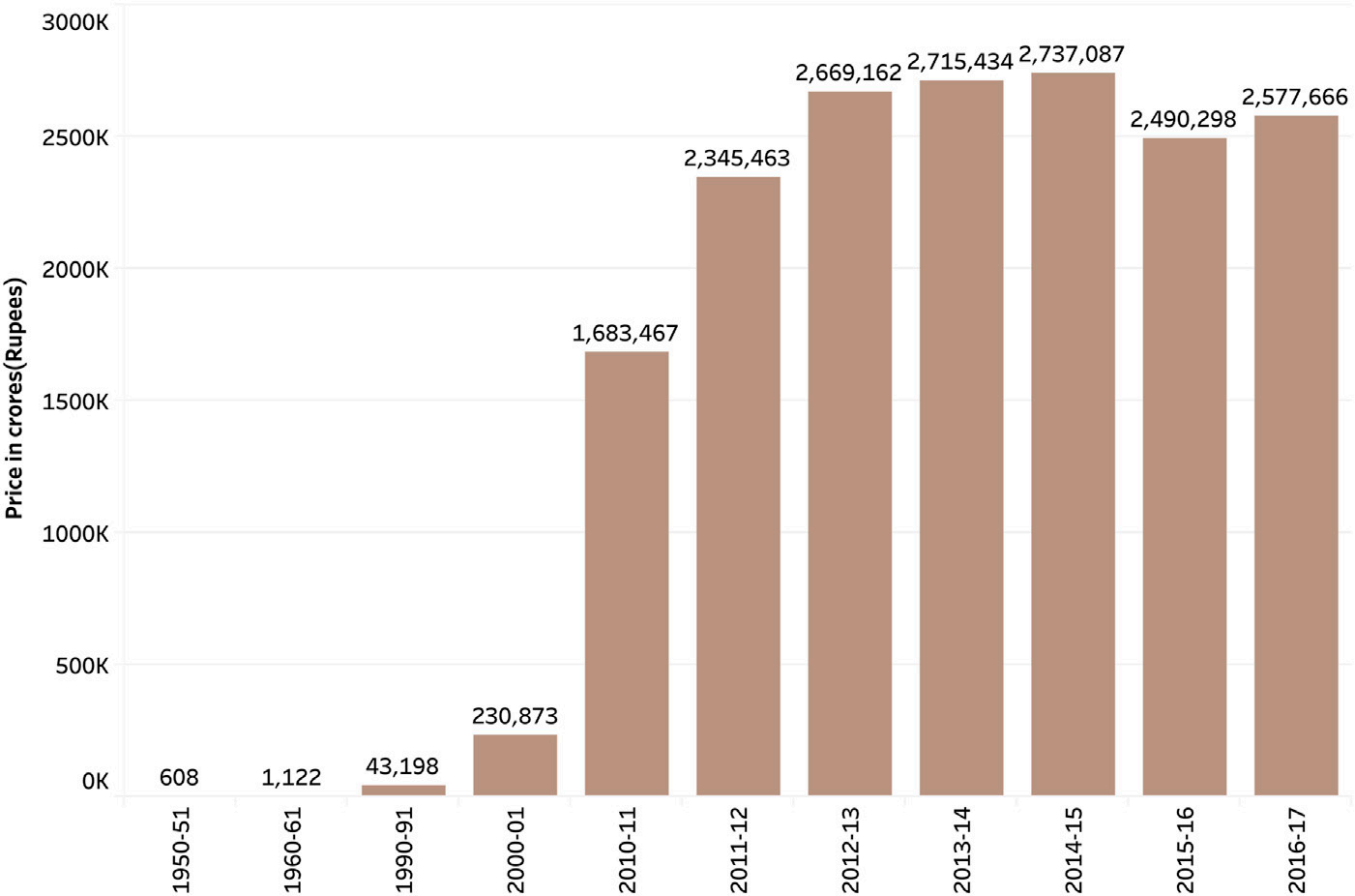
Imports of food items from India, Pocket book of Agricultural Statistics, India, 2017.

There has been a decline in the consumption of fruits and vegetables since the 1990s [18, 27]. During the time of globalization, many food items were on open general licensing, which implied that there is no requirement of permission from the government to import various food items. As a result, food-related imports of cereals and other products increased from 308,000 tons in the year 1990 to around 1,620,000 tons in the year 1999–2000. Edible oil imports increased from 526,000 tons in the year 1990 to 4,190,000 tons in 2002 [18]. Figures 2, 3 represent the overall imports and exports in crores in India from the year 1950–2017 [35].

### Development of the Food Industry in India

The development of the food industry in India reveals the impact of globalization on diets. The industry primarily consists of cereal, and sugar-based products such as bread, biscuits, cakes, pastries, pasta, cornflakes, and ready-to-eat/cook products. Out of these, bread and biscuits are produced in the largest quantity. Some cocoa products, soft drinks, alcoholic beverages, and mineral and packaged water are also crucial in the consumer industry. After packed tea and biscuits, soft drinks constitute the third largest industry. There is a large foreign investment in this. People have increased the consumption of fizzy drinks, pizzas, potato crisps, etc., thereby leading to increased sales of these products driving a demand. In addition to these, local products also flood the markets. Such local products mostly target the low-income groups and include potato chips and other fried items. The food processing industry is highly skewed towards cereal-based and sugar-based products more than meat, fish, poultry, milk, and vegetables [18]. In addition to all this, there are a lot of changes in the food supply system. Previously, the food supply system depended on local products from agriculture, livestock, and fisheries, which represent healthy sources of nutrition. But, this changed during the globalization of food systems [2].

### On the Health of the People

**FIGURE 4:**
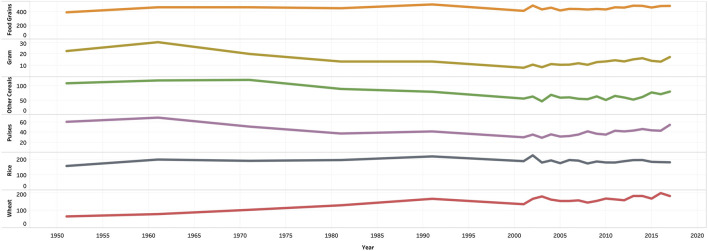
Per capita availability of food grains in India in terms of Gross Production Index-India, 2018.

**FIGURE 5:**
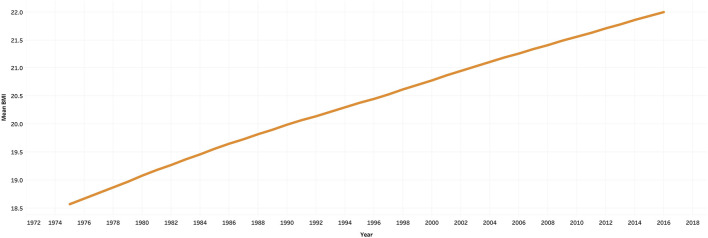
Mean BMI of women in India, ourworldindata, 2021.

**FIGURE 6:**
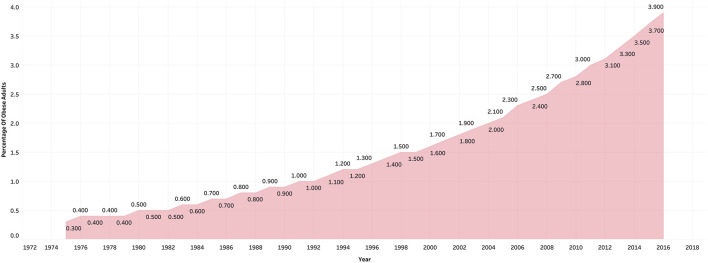
Percentage share of obese adults in India, ourworldindata, 2021.

The hike in the number of people who have diabetes and hypertension in India is due to the increase in processed food items [15]. The Comprehensive National Nutrition Survey reports that there were 166 million adults who were overweight and obese in India as of 2016. Other NCDs such as diabetes are being diagnosed in children, adolescents, and younger adults because of the increase in, physical inactivity, and faulty diet. Overall, India has seen its population moving from a predominantly underweight population to an overweight population in recent times [36]. Figure 4 shows the Gross Production Index number [37]. The Gross Production Index number is calculated by weighing the production quantity of each commodity by the 1989-91 average of international commodity prices and summed for each year. Further, to obtain the index, the aggregate of a given year is divided by the average aggregate for the base period [38]. This indicates an increasing trend in the consumption of rice and wheat as compared to the other cereals. Figure 5 shows the mean BMI of women in India [39]. From the figure, it is clear that obesity levels have been increasing over the years. Similarly, Figure 6 shows the percentage share of obese adults over the years which is also on an increasing trend [39]. Supplementary Material S1 represents the age-standardized mortality due to NCDs in India, which is also following a similar trend [40]. Similarly, Supplementary Material S2 shows the prevalence of diabetes in the Indian population over the years [41].

The unprocessed indigenous varieties of rice are protective against diabetes and hypertension and other diseases [42]. For instance, red rice, one of the traditional rice varieties, is a nutritious and medicinal food [42]. Red rice is also antifungal, anti-bacterial, anti-viral, anti-diarrheal, anti-inflammatory, antioxidant, antitumor, anti-thyroid, and anti-hyper cholesterolemic [42]. Most of the indigenous varieties are high in minerals and vitamins such as niacin, thiamine, iron, riboflavin, vitamin D, calcium, are high in fiber, and has less sugar content [42]. Those varieties also have a good amount of oryzanol, which could prevent cholesterol from building up in the body [42]. Also, another traditional staple food, millet, not only prevents the development of chronic non-communicable diseases but is also rich in micronutrients, components like lignans, and phytoestrogens, which helps in maintaining the hormonal balance in the body [15, 43]. A study found that the glycemic index of foxtail millet is much lower than that of rice [44]. Millets are good to consume as long as they are not hybridized or polished. This is where there is a need for significant measures to bring about behavioral changes at an individual level. This would include consumption of unpolished grains, particularly millets, reduction of junk food consumption, etc. For a holistic approach, the government has to be sensitive to the changing food preferences of the nation [45].

## Discussion

India might have to feed 394 million more people by the year 2050 because of the population increase [46]. Also, people are becoming less healthier with such a dietary shift as explained above. While taking care to feed the growing population, there has to be a consideration that the food has to be healthy.

In a study conducted to understand the relative benefits of grain usage, it was found that re-introduction of coarse grains like millets will make the food supply more nutritious, reduce resource demand and greenhouse gas emissions, and enhance climate resilience, without any reduction in calorie production and without necessitating more land for cultivation. But, this might require the conversion of some of the existing rice cultivated areas to millet cultivating areas. For the production of these, it is suggested that they might need more manure and power, but less fertilizer, labor, and machinery [47]. Currently, the production of millet is insufficient for the total millet supply [48]. But because PDS is a major source of food for the public, it would be advisable for the government to start backing the system of supplying millets. This initiation by the government can lead to a demanding drive for millets, thereby necessitating the farmers to start cultivating millets on large scales as they used to. Some states like Karnataka and Odisha have started state-level pilot programs for acquiring alternative cereals including millets from their farmers under the PDS system. This intervention can be tried in other states as well [44]. Also, the Minister of Agriculture and Farmers’ Welfare had organized a consultative committee to promote the cultivation of millets, based on the evidence that the production of millets can help in nutritional security, sustainability, and prevent malnutrition. The year 2018 was declared the “National year of millets” by the Ministry [21]. Such measures would, in turn, alleviate malnutrition, improve the health of the people, and it can lead to environmental sustainability. For the food-related health issues that India is facing now, the only solution is either to go back to the consumption of indigenous varieties of rice, which were unpolished, or to start consuming millets and traditional grains and to reduce the consumption of junk food.

### Limitations

This review paper has not covered the statistics and the numbers related to the issues discussed in detail.
