# Corrigendum: What Is the Modern Human Eating? Dietary Transition of the Age-Old to the Modern Man of India

**DOI:** 10.3389/phrs.2024.1607464

**Published:** 2024-05-29

**Authors:** Daisy A. John, Giridhara R. Babu

**Affiliations:** ^1^ Indian Institute of Public Health, Bengaluru, India; ^2^ Department of Population Medicine, College of Medicine, QU Health, Qatar University, Doha, Qatar

**Keywords:** food transition, processed food, globalization, staples, behavioral change

In the published article, there was an error in **Affiliation** of Giridhara R. Babu. Instead of “Indian Institute of Public Health, Bengaluru, India”, it should be “Department of Population Medicine, College of Medicine, QU Health, Qatar University, Doha, Qatar.”

The authors apologize for this error and state that this does not change the scientific conclusions of the article in any way. The original article has been updated.

