# Supplementary figures and images for: What is the Modern Human Eating? Dietary Transition of the Age-Old to the Modern Man of India
Source: Public Health Rev. 2022 Mar 18;43:1604058. doi: 10.3389/phrs.2022.1604058 (PMC8971190; doi:10.3389/phrs.2022.1604058)

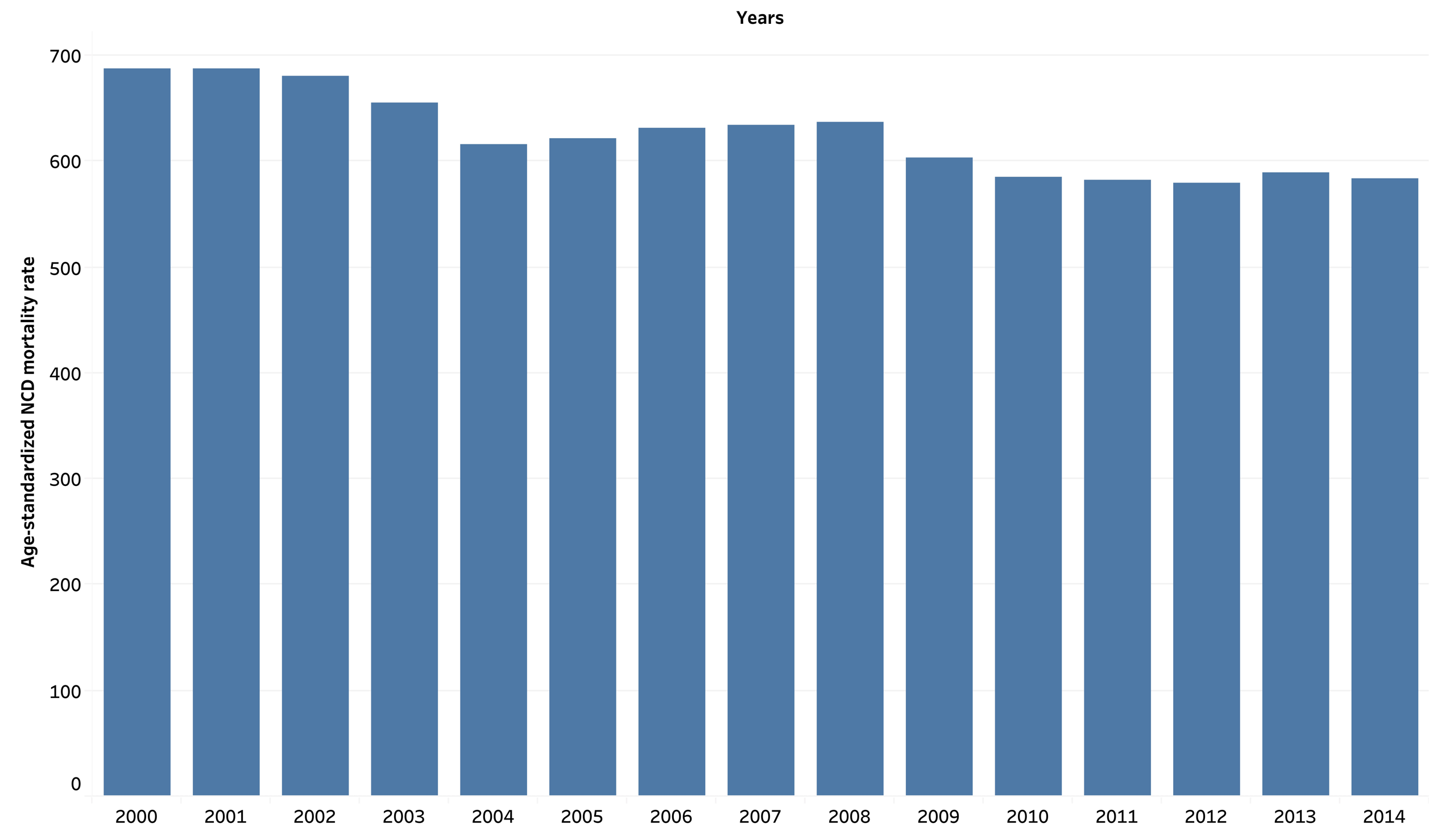

Supplement: Supplementary file 1 [file Image1.JPEG]

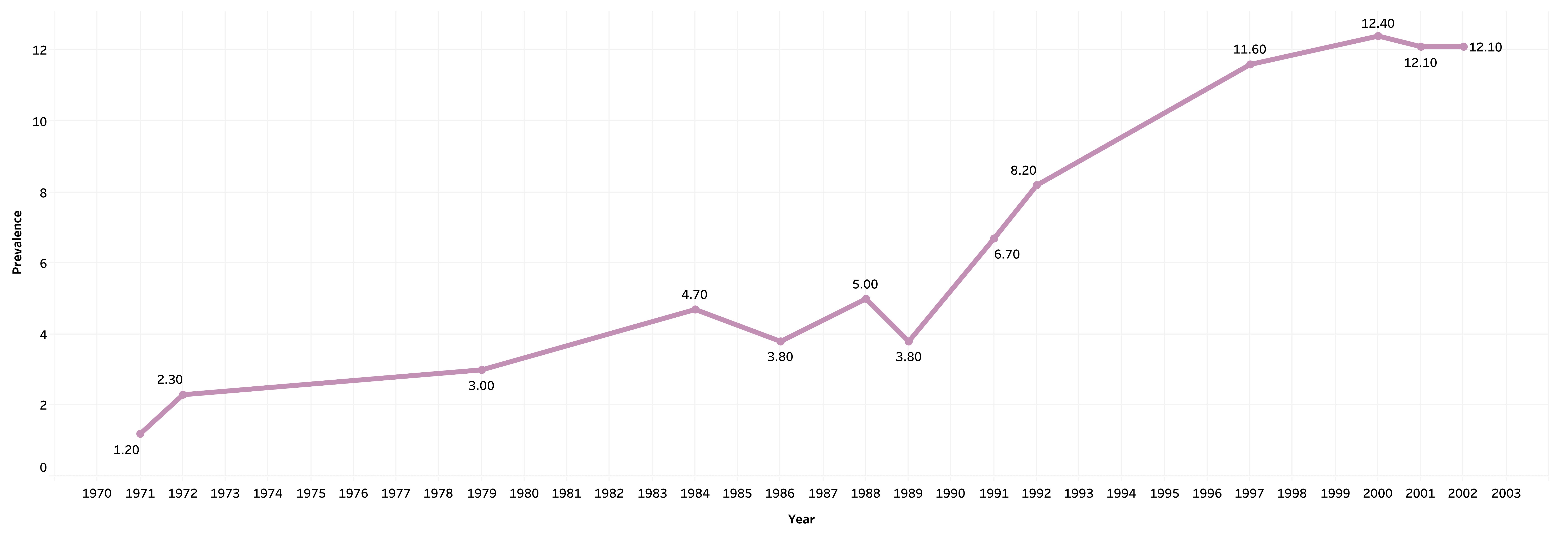

Supplement: Supplementary file 2 [file Image2.JPEG]
